# Predictors and Prognostic Impact of Left Ventricular Ejection Fraction Recovery after Impella-Supported Percutaneous Coronary Interventions in Acute Myocardial Infarction

**DOI:** 10.3390/jpm12101576

**Published:** 2022-09-24

**Authors:** Federico Marin, Michele Pighi, Federico Zucchelli, Alessandro Ruzzarin, Giulio Russo, Cristina Aurigemma, Enrico Romagnoli, Valeria Ferrero, Anna Piccoli, Roberto Scarsini, Gabriele Pesarini, Carlo Trani, Francesco Burzotta, Flavio Luciano Ribichini

**Affiliations:** 1 Division of Cardiology, Department of Medicine, University of Verona, 37126 Verona, Italy; 2 Department of Cardiovascular Medicine, Fondazione Policlinico Universitario A. Gemelli IRCCS, 00168 Roma, Italy; 3 Department of Cardiovascular and Pulmonary Sciences, Università Cattolica del Sacro Cuore, 00168 Roma, Italy

**Keywords:** hemodynamic support, percutaneous coronary intervention, acute coronary syndrome

## Abstract

Aim: The aim of our study is to assess the predictors and the prognostic role of left ventricle ejection fraction (LVEF) recovery after Impella-supported percutaneous coronary intervention (PCI) in patients presenting with acute myocardial infarction (AMI). Methods: This retrospective, observational study included patients admitted for AMI who underwent Impella-supported PCI in two Italian high-volume cardiac catheterization laboratories. Only patients who underwent an echocardiographic assessment of left ventricle ejection fraction (LVEF) before the procedure (acute LVEF) and during follow-up (follow-up LVEF) were included in the present analysis. Patients with a baseline LVEF ≥40% were excluded from the present analysis. LVEF recovery was calculated as the difference between follow-up LVEF and acute LVEF. A delta ≥5% was considered significant and was used to define the responder group. Results: From April 2007 to December 2020, 64 consecutive patients were included in our study. A total of 55 patients (86%) received hemodynamic support with Impella 2.5, and 9 patients (14%) with Impella CP. Median LVEF at follow-up was significantly higher compared to baseline (36% (30–42) vs. 30% (24–33), *p* < 0.001). Based on LVEF recovery, 37 patients (57.8%) were deemed responders. According to multivariate analysis, complete functional revascularization was an independent predictor of a significant EF recovery (OR: 0.159; 95% CI: 0.038–0.668; *p* = 0.012). At three-year follow-up, lack of LVEF recovery was the only predictor of mortality (HR: 5.315; 95% CI: 1.100–25.676; *p* = 0.038). Conclusions: Functional complete revascularization is an independent predictor of the recovery of LVEF in patients presenting with AMI who underwent Impella-supported PCI. The recovery of LV function is associated with improved prognosis and could be used to stratify the risk of future events at long-term follow-up.

## 1. Introduction

Acute myocardial infarction (AMI) represents one of the leading causes of morbidity and mortality worldwide, and emergency coronary surgery is seldom offered as an alternative. Moreover, data suggest that the spectrum of comorbidities and the complexity of coronary anatomy of patients presenting to catheterization laboratories has increased [1]. Patients with hemodynamic compromise and complex coronary artery disease are increasingly referred to percutaneous coronary interventions (PCI) [2], which frequently require extensive atherectomy, repetitive and prolonged balloon inflations, complex stenting techniques, and high contrast volumes. Although multiple definitions of high-risk PCI patients have been proposed, the features identifying these patients are mainly related to three clinical areas: 1) patient risk factors and comorbidities, 2) location of the disease and complexity of coronary anatomy, and 3) hemodynamic clinical status [3]. 

An emergent strategy to facilitate PCI in this cohort of patients is pre-emptive mechanical cardiac support (MCS) [4]. The aim of so-called MCS-protected PCI is to guarantee adequate hemodynamic support during the critical steps of PCI in order to improve the cardiac output and maintain adequate coronary and systemic perfusion while minimizing the risk of ischemia. The Impella (Abiomed, Danvers, MA, USA) is one of the MCS devices available in catheterization laboratories. It consists of a percutaneous microaxial flow pump placed into the left ventricle (LV), which increases the cardiac output and unloads the LV. Various devices have been developed; the Impella 2.5 system provides a maximum output of 2.5 l/min, and the Impella CP system provides a maximum output of as much as 3.7 l/minL/minute. The PROTECT I study demonstrated the safety and feasibility of Impella 2.5 in the context of high-risk PCI in a small cohort of 20 patients [5]. The PROTECT II trial randomized patients to receive circulatory support with IABP or Impella 2.5. The study was discontinued due to futility based on 30-day outcomes; nevertheless, a trend of improved outcomes was observed among Impella-supported patients at the 90-day follow-up [6]. Data from real-word registries have shown that Impella may facilitate more extensive revascularization in elective high-risk PCI, improving LV recovery and survival [7]. However, the majority of studies have excluded patients presenting with acute myocardial infarction undergoing urgent revascularization. 

The purpose of the present study is to assess the predictors and the prognostic impact of left ventricle ejection fraction (LVEF) recovery after Impella-supported revascularization in patients presenting with AMI, including patients whose status is complicated by cardiogenic shock (CS) or presenting with out-of-hospital cardiac arrest (OOHCA).

## 2. Methods

### 2.1. Study Design

The present analysis was focused on a series of consecutive patients presenting with AMI, including patients presenting with OOHCA or those whose status was complicated by CS who underwent urgent Impella-protected PCI at the cardiac catheterization laboratories of the Verona University Hospital and the Gemelli University Hospital. Indications for urgent revascularization included ST-elevation myocardial infarction or high-risk non-ST-elevation myocardial infarction in the presence hemodynamic instability, recurrent or refractory chest pain despite medical treatment, life-threatening arrhythmias, or the presence of ST-segment depression > 1 mm in ≥ 6 leads, in addition to ST-segment elevation in aVR and/or V1. According to hospital practice, the need for Impella support was assessed based on a collegial heart-team decision in the catheterization laboratory based on a combination of criteria, including clinical and hemodynamic presentation, the severity of left ventricular dysfunction, and the extension and complexity of coronary artery disease. 

**Figure 1:**
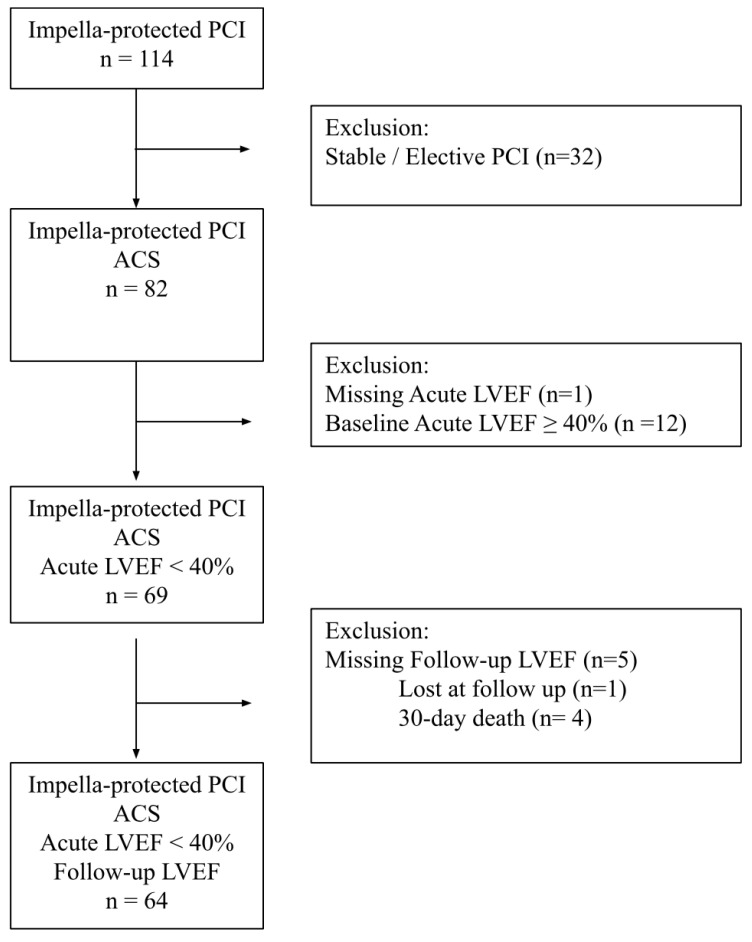
Flow chart of the study protocol.

In order to assess the changes in the LVEF after Impella-supported PCI, the present study included only patients with at least two echocardiographic assessments, including one before the procedure (acute LVEF) and at least one at follow-up (follow-up LVEF). Patients with a baseline LVEF above 40% were excluded. A flow chart of the study protocol is reported in Figure 1. 

Clinical characteristics, comorbidities, and cardiac medications were collected prospectively in a dedicated database. Follow-up data were obtained through medical interviews and electronic patient records. Preoperative risk assessment was performed using EuroSCORE II scores [8]. Visual coronary angiography was the method of choice to assess stenosis grade; a stenosis ≥ 70% was considered significant (≥ 50% in the case of the left main coronary artery). For all patients, the synergy between percutaneous coronary intervention with TAXUS and cardiac surgery (SYNTAX) score was calculated before and after the procedure [9]. Additionally, the myocardium in jeopardy before and after PCI was quantified using the British Cardiovascular Intervention Society (BCIS) jeopardy score (JS) algorithm [10]. The revascularization index (RI) was assessed for all patients (RI = (BCIS-JS pre-BCIS-JS post)/BCIS-JS pre) [11]. Functional complete revascularization was defined as successful reperfusion of all the ischemic myocardial territories, in contrast to areas of old infarction with no viable myocardium, which was not reperfused [12]. The median follow-up of the population was three years.

### 2.2. Impella-Protected PCI

Impella 2.5 or Impella CP was implanted upstream of the PCI in all patients. According to manufacturer recommendations, the devices were implanted via a percutaneous transfemoral approach. Peripheral angiography was recommended as guidance to place the devices in consideration of anatomic feasibility (i.e., the presence of high tortuosity or atherosclerotic burden was the criterion to consider the contralateral iliac–femoral axis to position the Impella device). The insertion of a 6–8 Fr sheath followed the femoral artery stick. The “preclosure’’ technique was performed through suture-based hemostatic devices (Perclose Proglide, Abbot Vascular Devices, CA, USA). After the insertion of a dedicated sheath, a diagnostic catheter (6 Fr) was advanced into the left ventricle to position an extra-support guidewire into the left ventricle (LV). Then, the Impella catheter was advanced over the guidewire through the aortic valve into the LV and activated after removing the guidewire.

According to revascularization guidelines, PCI was performed by the radial or, in patients with unsuitable access, by a contralateral femoral approach using 6–8 Fr catheters.

Drug-eluting stent implantation was the principal PCI technique applied, and lesion debulking with Rotablator as an adjunctive device was used for severely calcified coronary segments.

At the end of the PCI, the speed of the Impella was gradually decreased, and the device was removed prior to confirmation of hemodynamic stability. In the case of hemodynamic instability or need for ECMO positioning, the Impella was left on site. Access-artery angiography was performed to confirm hemostasis and to rule out vascular complications, whereas mechanical hemostasis failures were managed by manual compression and compressive bandaging.

Heparin (weight-adjusted) was administered intravenously in all patients, and the following boluses were administered to maintain activated clotting time between 250 and 300s.

Before and after PCI, drugs were administered according to accepted guidelines and established practice standards, including dual antiplatelet therapy, ace inhibitors, proton pump inhibitors (PPIs), beta blockers, and statins. 

### 2.3. Procedural and Clinical Outcome Assessment

For the present study, clinical records were carefully evaluated. Based on biochemical analyses, troponin I or T and creatine kinase MB were used as biomarkers to quantify myocardial damage. The access site or bleeding complications were assessed according to Bleeding Academic Research Consortium (BARC) guidelines [13]. Clinical outcomes were collected through the institutional electronic medical record system. If necessary, office visits or telephone contact were conducted to confirm the clinical outcome of patients and ensure data completeness. 

### 2.4. Echocardiographic Assessment

Echocardiographic examinations were performed by expert operators. Data were retrospectively collected from medical notes. LVEF before the procedure (acute LVEF) was obtained from the echocardiographic assessment systematically performed before the index procedure. LVFE at follow-up (follow-up LVEF) was obtained from the echocardiogram performed as part of routine clinical practice at both institutions. LVEF recovery was calculated as the difference between follow-up LVEF and acute LVEF. Based on previous data, an EF recovery of 5% was considered a significant improvement of LV function [14,15] and was used to define the responder group. 

### 2.5. Statistical Analysis 

After verifying normal distribution by Shapiro–Wilk test, variables were expressed as mean and standard deviation (SD) or as median and interquartile range (IQR) as appropriate. Frequencies were compared using the chi-square test or Fisher’s exact test as appropriate. Continuous variables were compared using the t-test or Mann–Whitney test if not normally distributed. Univariate and multivariate logistic regression analyses were conducted to assess baseline predictors of improvement of EF. Survival was displayed using Kaplan–Meier curves and compared using the log-rank test. The univariable and multivariable Cox proportional hazards models were used to estimate hazard ratios and 95% confidence intervals. Statistical analysis was performed using SPSS 26.0 (SPSS, Inc., Chicago, Illinois, USA), and a *p*-value < 0.05 was considered statistically significant. 

## 3. Results

### 3.1. Population

From April 2007 to December 2020, 64 consecutive patients presenting with AMI who underwent Impella-supported PCI were included in our study. Baseline clinical characteristics are reported in Table 1. The median age of the patients was 73 (66–81) years, and 54 (84.4%) were men. The most frequent clinical presentation was non-ST-elevation myocardial infarction (NSTEMI) (40, 62.5%), whereas 24 (37.5%) patients presented with ST-elevation myocardial infarction (STEMI). Fifteen (26.3%) patients presented with concomitant CS, and five (7.8%) were resuscitated from OOHCA. 

Procedural and angiographic characteristics of the study population are reported in Table 2. All patients underwent urgent coronary angiography. Multivessel coronary artery disease was reported in 58 patients (95.1%), 30 patients (49.2%) presented with severe stenosis of the left main stem (LMS), and 48 patients (78.7%) presented with left anterior descending artery (LAD). All patients underwent Impella-supported PCI. A total of 55 patients (85.9%) received hemodynamic support with Impella 2.5, and 9 patients (14%) received hemodynamic support with Impella CP. In four patients, an Impella was used in combination with venoarterial extracorporeal membrane oxygenation (ECMO) due to refractory cardiogenic shock at presentation. PCI was performed via femoral artery access in 56 (88%) patients. Twelve patients (15%) received rotational atherectomy for heavily calcified lesions. BCIS jeopardy score was significantly reduced after PCI compared to baseline (from 12 (9–12) to 2 (0–4), *p* < 0.05). Functional complete revascularization was achieved in 41 cases (64%). The median duration of Impella support was 150 min (115–4206). Successful hemostasis was achieved by inserting a double preimplanted Perclose Proglide in 59.7%, Prostar XL in 17.7%, and three patients underwent surgical closure. 

According to the Bleeding Academic Research Consortium (BARC) scale, we reported six minor bleeding cases (BARC Type I-II) not requiring a specific treatment. A total of 10 patients presented with major bleeding, of which four presented with BARC type IIIa and 4 had bleeding that required interventions (2 cases of bleeding at the cannula insertion during venoarterial ECMO support combined with Impella device and 2 cases of coronary perforation during PCI requiring urgent pericardiocentesis). No major vascular complications were reported as shown in Table 3.

### 3.2. Recovery of LV Function 

According to the study inclusion criteria, all patients underwent an echocardiogram as part of routine clinical practice at a median of 6 months (1–12 months) after the index procedure. 

An EF recovery of at least 5% was observed in 37 patients (57.8%) (responder group). A comparison of baseline characteristics between the responder and the non-responder group is reported in Table 1. The proportion of patients achieving complete revascularization at the end of the procedure was significantly higher in the responder group compared to the non-responders (81.1% vs. 40.7%, *p* = 0.013). Accordingly, the revascularization index (RI) was significantly higher (0.83 vs. 0.66, *p* = 0.025), and the post-PCI BCIS-JS was significantly lower in the responder group (1.99 vs. 4, *p* = 0.022). Medical therapy was similar between the two groups.

Univariate analysis showed that lower LVEF at baseline (odds ratio (OR): 0.915; 95% confidence interval (CI): 0.838–0.999; *p* = 0.048), functional complete revascularization (OR: 5.5; 95% CI: 1.4–21; *p* = 0.013), higher revascularization index (OR: 13.5; 95% CI: 1.2–143.4; *p* = 0.031), and lower post-PCI BCIS-JS (OR: 0.78; 95% CI: 0.6–0.96; *p* = 0.023) were predictors of LVEF recovery at follow-up, as shown in Table 4. To overcome the collinearity between the variables, three models were designed, confirming that complete functional revascularization is an independent predictor of LVEF recovery at follow-up, as well as revascularization index and post PCI BCIS-JS. 

### 3.3. Clinical Outcomes

At the three-year follow-up, the overall mortality rate was 14.1%. Patients with an EF recovery of at least 5% (responder group) had lower mortality compared to the group in which LVEF remained stable (5.4% vs. 25.9%; *p* = 0.029). Univariate Cox regression analysis showed that improvement in LVEF at follow-up of at least 5% and LVEF at follow-up were the only predictors of three-year mortality (HR 5.3; 95% CI. 1.1–25.7, *p* = 0.038) as shown in Table 5. 

**Figure 2:**
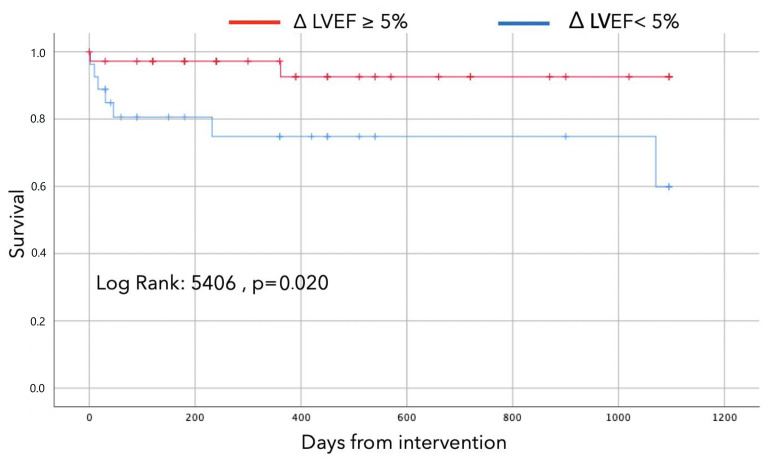
Survival curves according to improvement in LVEF at follow-up of at least 5%.

Kaplan–Meier curves are displayed in Figure 2 and demonstrate early separation and persistent divergence at three-year follow-up among patients who experienced LVEF recovery compared to those in which LVEF did not improve (Log-rank 5,40, *p*-value 0.02). 

## 4. Discussion

The present observational study reports on the predictors and the prognostic role of LVEF recovery after Impella-supported revascularization in patients presenting with AMI with acute myocardial infarction at two experienced Italian centers. 

The main findings of the present analysis are:

1. Functional complete revascularization was an independent predictor of an EF recovery of at least 5% at follow-up; and
2. An EF recovery of 5% was associated with a significant survival benefit.

Our results are consistent with a growing body of evidence demonstrating that protected PCI with Impella is associated with improved LVEF and heart failure symptoms. Maini et al. reported a significant increase in LVEF upon discharge (from 31.15% to 36.14%, *p* < 0.0001) in a cohort of 175 consecutive patients who underwent high-risk PCI with prophylactic support of the Impella 2.5. Interestingly, improvement in LVEF was greater in patients who underwent a PCI on the last remaining patent conduit or in multivessel disease [16]. Similar findings were recently confirmed by an interim analysis of the Restore EF study, an ongoing multicenter, prospective, single-arm study that enrolled 193 consecutive qualified patients who underwent a protected PCI procedure with Impella. The analysis showed a significant LVEF improvement from baseline to 90-day follow-up (31% to 45% *p* < 0.0001), a significant reduction in heart failure symptoms with an 80% reduction in New York Heart Association (NYHA) classification III/IV at follow-up (54% to 11% *p* < 0.001), and a significant reduction in anginal symptoms with 99% reduction in Canadian Cardiovascular Society (CCS) classification III/IV at follow-up (70% to 1% *p* < 0.0001). 

Our group previously showed that protected PCI with Impella is associated with LVEF improvement in complex, high-risk patients at 90-day follow-up (27% vs. 33%, *p* < 0.001), and complete revascularization is associated with increased LVEF and survival [7]. The present analysis extended those findings in patients with acute myocardial infarction undergoing urgent PCI, who were excluded from our previous analysis, and extended the results at a longer follow-up time. 

The role of complete revascularization in patients with myocardial infarction is still debated. Urgent PCI of the infarct-related artery is imperative; however, the management of non-culprit arteries is controversial. Although multivessel PCI may reduce the burden of global myocardial ischemia and improve myocardial function, on the other hand, it may cause harm due to procedural complications, increased procedural time and contrast volume, and possible ischemia in different territories. In patients with multivessel disease and AMI without CS, the previous trials, i.e., DANAMI-3-PRIMULTI (complete revascularization versus treatment of the culprit lesion only in patients with ST segment elevation myocardial infarction and multivessel disease; *n* = 627) [17], PRAMI (randomized trial of preventive angioplasty in myocardial infarction; *n* = 465) [18], and CvLPRIT (randomized trial of complete versus lesion-only revascularization in patients undergoing primary coronary intervention for STEMI and multivessel disease; *n* = 296 ), have suggested potential benefits of complete revascularization [19]. However, in patients with CS, the randomized, multicenter, large-scale CULPRIT-SHOCK trial (PCI strategies in patients with acute myocardial infarction and cardiogenic shock; *n* = 706) [20,21] showed a better outcome in the cohort who initially underwent PCI of the culprit lesion only, as compared with those who underwent multivessel PCI. Notably, an MCS device was used only in 28% of patients. Another caveat of the study is the management of non-infarct related chronic total occlusions (CTO), which were found in almost a quarter of patients presenting with AMI complicated by CS. A prespecified analysis of the trial showed that a strategy of culprit-lesion-only PCI was associated with lower rates of death or renal replacement therapy at 30-day follow-up in patients with and without CTO [22]. It is well-known that CTO-PCI increases procedural risk, as well as the time of the procedure and the amount of contrast, and the prognostic benefits are still debated. In our opinion, the systematic revascularization of CTO, in particular if non-infarct related, irrespective of the viability of the target myocardium (complete anatomical revascularization), is not justified, is potentially harmful in the setting of AMI, and could potentially dilute the benefit of achieving complete functional revascularization in this subset of patients. 

The RECOVER IV randomized controlled trial (RCT) will assess whether Impella pre-PCI is superior to PCI without Impella in patients with AMI cardiogenic shock. RECOVER IV will be a prospective, two-arm trial. Patients will be randomized to receive either Impella pre-PCI or other treatment protocols, which may include any kind of non-Impella circulatory support. In our experience, Impella support during high-risk PCI can guarantee coronary and systemic perfusion during periods of myocardial ischemia during prolonged or repeated balloon inflations or atherectomy runs, allowing for complete functional revascularization, which could improve ventricular function and long-term prognosis. 

## 5. Limitations

We acknowledge several limitations of our analysis. The present work is a retrospective, observational study, collecting data from two large and experienced Italian centers, and patient selection was based on local heart-team decisions. The presented population was enrolled during the learning curve in the management of Impella. Another limitation of our study is the exclusion of patients who did not under an echocardiogram both before (*n* = 1) and during the follow-up (*n* = 5). The reason for this inclusion criterion was to assess the changes in LV function after revascularization, which, unfortunately, led to the exclusion of patients who died within 30 days or were lost to follow-up (*n* = 4, *n* = 1, respectively). This might also explain the relatively low 3-year mortality of our population (14.1%) compared to our series. Moreover, our analysis did not include a control group. Considering such aspects, the present study results should be considered as hypothesis-generating. The results of the ongoing randomized trial will be key in providing further insights on the subject. 

## 6. Conclusions

Functional complete revascularization is an independent predictor of recovery of LV function in patients presenting with AMI who underwent Impella-supported PCI. The recovery of LV function is associated with improved prognosis and could be used to stratify the risk of future events upon long-term follow-up. 

## Figures and Tables

**Table 1:** Baseline clinical characteristics of the study population.

	Overall (*n* = 64)	ΔLV EF < 5% (*n* = 27, 42.2%)	ΔLVEF > 5% (*n* = 37, 57.8%)	*p*-Value
Age (years)	73 (66–81)	72 (66–81)	73 (66–81)	0.935
Sex, male (*n*/%)	54 (84.4)	24 (88.9)	30 (81.1)	0.498
BMI	26.2 (23.2– 29.4)	25.06 (23.15–29.4)	26.77 (23.7–29.4)	0.749
Cardiovascular risk factors				
Hypertension (*n*/%)	44 (69.8)	17 (65.49)	27 (73)	0.583
Dyslipidemia (*n*/%)	32 (51.6)	14 (56)	18 (48.6)	0.613
Diabetes (*n*/%)	24 (38.7)	10 (40)	14 (37.8)	1
Smoking (*n*/%)	15 (24.6)	8 (33.3)	7 (18.9)	0.235
Familial history of CAD (*n*/%)	12 (19.4)	7 (28)	5 (13.5)	0.198
CKD (*n*/%)	20 (31.5)	11 (40.7)	9 (24.3)	0.379
Previous stroke (*n*/%)	4 (6.3)	2 (7.4)	2 (5.4)	1
PAD (*n*/%)	16 (25.4)	7 (26.9)	9 (24.3)	1
Past cardiac history (*n*/%)				
Previous ACS (*n*/%)	17 (26.6)	8 (29.6)	9 (24.3)	0.776
Previous PCI (*n*/%)	9 (14.3)	4 (15.4)	5 (13.5)	1
Previous CABG (*n*/%)	9 (14.1)	5 (18.5)	4 (10.8)	0.475
Clinical presentation (*n*/%)				
OHCA (*n*/%)	5 (7.8)	2 (7.4)	3 (8.1)	1
Cardiogenic shock°	15 (26.3)	5 (19.2)	10 (32.3)	0.368
STEMI (*n*/%)	24 (37.5)	9 (33.3)	15 (40.5)	0.609
NSTEMI (*n*/%)	40 (62.5)	18 (66.7)	22 (59.5)	0.609
Delay > 24 h (*n*/%)	18 (28.1)	6 (22.2)	12 (32.4)	0.659
Preprocedural MOF (*n*/%)	0 (0)	0 (0)	0 (0)	//
Preprocedural PAS (mmHg)	109.4 ± 19.2	108.8 ± 15.9	109.8 ± 22.0	0.904
Preprocedural PAD (mmHg)	65.6 ± 13.0	68.9 ± 13.6	62.9 ± 12.3	0.230
Preprocedural HR (bpm)	87.1 ± 23.6	92.5 ± 26.4	82.4 ± 21.7	0.466
EKG presentation				
R waves (*n*/%)	36 (56.3)	12 (44.4)	24 (65.8)	0.305
ST segment deviation (*n*/%)	31 (48.4)	12 (44.4)	19 (48.7)	0.745
LBBB (*n*/%)	19 (29.7)	14 (52)	9(23.5)	0.219
RBBB (*n*/%)	9 (14.1)	0 (0)	9 (24.3)	0.286
Echocardiography				
LVEDVi (ml/mq)	89.4 ± 20.9	88.7 ± 26.7	90 ± 16.5	0.901
LVEF at baseline (%)	30 (24.3–33)	31 [28–33.5]	28 [22–33]	0.102
WMSI (*n*)	2.06 (1.97–2.30)	2.03 [2–2.06]	2.18 [1.97–2.45]	0.462
SPAP (mmHg)	35 (18–50)	40 [20.5–57.5]	32.5 [19–48.5]	0.432
RV dysfunction	13 (20.3)	2 (7.4)	11 (29.7)	0.358
LVEF at 6 months (%)	36.2 ± 9.32	30.0 ± 5.50	40.6 ± 9.07	**<0.001**
Laboratory test				
CK-mb peak (U/L)	25 [10–87.8]	27.5 [12.3–125.3]	20.5 [6.8–112.9]	0.561
Creatinine admission (mg/dL)	1.1 [0.9–1.33]	1.1 [0.9–1.22]	1.15 [1.03–1.4]	0.413
Creatinine at 48 h (mg/dL)	1.35 [1.0–1.88]	1.49 [1.02–1.90]	1.3 [0.99–1.80]	0.539
Lactate, admission (mmol/)	2.3 [1.3–9.4]	2.1 [1.25–2.95]	4 [1.35–10.2]	0.397

BMI = body mass index; CAD = coronary artery disease; CKD = chronic kidney disease, abnormalities of kidney function (decreased glomerular filtration rate < 60 mL/min/1.73m2) or kidney structure (e.g., kidney transplantation); PAD = peripheral artery disease; ACS = acute coronary syndrome; PCI = percutaneous coronary interventions; CABG = coronary artery bypass graft; OHCA = out-of-hospital cardiac arrest; STEMI = ST-elevation myocardial infarction; NSTEMI = non-ST-elevation myocardial infarction; LBBB = left bundle branch block; RBBB = right bundle branch block; LVEDVi = left ventricular end diastolic volume; WMSI = wall motion score index; SPAP = systolic pulmonary artery pressure; RV = right ventricle; CK-MB = creatine kinase-MB.

**Table 2:** Procedural and angiographic characteristics of the study population.

	Overall (*n* = 64)	ΔLV EF < 5% (*n* = 27)	ΔLVEF > 5% (*n* = 37)	*p*-Value
Circulatory support				
Impella 2.5 (*n*/%)	55 (85.9)	23 (85.2)	32 (86.5)	1
Impella CP (*n*/%)	9 (14.1)	4 (14.8)	5 (13.5)	1
Duration of support (min)	150 [115–4206]	127 [99–2945.5]	163 [123–3080]	0.351
Vasoactive drugs * (*n*/%)	24 (37.5)	10 (37)	14 (37.8)	1
Mechanical ventilation (*n*/%)	20 (31.2)	8 (29.6)	12 (32.4)	1
Angiographic and Procedural characteristics				
Multivessel disease (*n*/%)	58 (95.1)	24 (96)	34 (94.4)	1
LM disease (*n*/%)	30 (49.2)	12 (48)	18 (50)	1
LAD disease (*n*/%)	48 (78.7)	20 (80)	28 (77.8)	1
LCX disease (*n*/%)	27 (44.3)	10 (40)	17 (47.2)	0.610
RCA disease (*n*/%)	20 (32.8)	5 (20)	15 (41.7)	0.100
BCIS JS pre-PCI	12 (9–12)	12 [10–2]	10 [8–12]	0.218
BCIS JS post-PCI	2 (0–4)	4 [2–6]	1.99 [0–4]	**0.022**
Revascularization index	0.67 [0.55–1]	0.66 [0.5–0.82]	0.83 [0.66–1]	**0.025**
Functional complete revascularization	28 (63.6)	8 (42.1)	20 (80)	**0.013**
Euroscore II	8.5 [5.5–17.2]	5.82 [5.51–13.35]	12.02 [5.46–17.67]	0.412
Syntax score	31.5 ± 11.57	33.31 ± 10.88	30.40 ± 12.0	0.369
1 vessel disease (*n*/%)	5 (8.3%)	2 (8%)	3 (8.69%)	0.978
2 vessel disease (*n*/%)	14 (23.3%)	6 (24%)	8 (22.9%)	
3 vessel disease (*n*/%)	41 (68.3%)	17 (68%)	24 (68.6%)	
Rotational atherectomy (*n*/%)	12 (20)	4 (16)	8 (22.9)	0.745
Stent length (mm)	47.32 ± 26.66	46.91 ± 17.25	47.59 ± 31.79	0.949
Contrast dye (mL)	227.27 ± 86.54	220.91 ± 94.38	232.5 ± 83.56	0.740
Procedure time (min), mean ± SD	124 ± 75	109.38 ± 45.72	135.26 ± 43.99	0.118
**Closure device**				
ProGlide (*n*/%)	5 (8.1)	1 (4)	4 (10.8)	0.640
ProStar (*n*/%)	11 (17.7)	6 (24)	5 (13.5)	0.326
Dual Perclose (*n*/%)	32 (51.6)	9 (36)	23 (62.2)	0.069
Surgical (*n*/%)	3 (4.8)	1 (4)	2 (5.4)	1
Medical therapy at follow-up				
Beta blockers	61 (95.3)	27 (100)	34 (91.9)	0.2567
ACE-i	50 (78.1)	20 (74)	30 (81)	0.1905
Diuretics	61 (95.3)	27 (100)	34 (92)	0.2567
MRA	63 (98.4)	27 (100)	36 (97.2)	1

* norepinephrine, epinephrine, dopamine, dobutamine; alone or in combination; ACE-i = angiotensin-converting enzyme inhibitor; LM = left main; LAD = left anterior descending coronary artery; LCX = left circumflex artery; MRA = mineralocorticoid receptor antagonist; RCA = right coronary artery; BCIS-JS = British Cardiovascular Intervention Society myocardial jeopardy score [0–12].

**Table 3:** Complications and outcomes in the study population.

	Overall (*n* = 64)	ΔLV EF < 5% (*n* = 27)	ΔLVEF > 5% (*n* = 37)	*p*-Value
Intraprocedural death	0 (0)	0 (0)	0 (0)	-
Intraprocedural complications	4 (6.3)	2 (7.4)	2 (5.4)	1
Bleeding	13 (20.31)	5	9	
BARC 1 *	2 (3.1)	2 (7.4)	0	-
BARC 2	4 (6.3)	3 (11.1)	1 (2.7)	0.219
BARC 3A	4 (6.3)	0	4 (10.8)	-
BARC 3B	4 (6.3)	0	4 (10.8)	-
BARC 4	0	-	-	-
BARC 5	0	-	-	-
Compartment syndrome	3 (4.7)	-	3 (6.8)	-
Death at 3 year follow-up	9 (14.1)	7 (25.9)	2 (5.4)	**0.021**

* According to the definition of the Bleeding Academic Research Consortium (BARC).

**Table 4:** Univariate and multivariate analysis of the predictors of LVEF recovery at follow-up.

	Univariate Analysis		Model A		Model B		Model C	
	*p*-Value	OR (95% C.I.)	*p*-Value	OR (95% C.I.)	*p*-Value	OR (95% C.I.)	*p*-Value	OR (95% C.I.)
Age (year)	0.806	1.01 (0.69–1.05)						
Sex	0.401	1.87 (0.44–8.00)						
BMI	0.916	1.01 (0.89–1.15)						
OHCA	0.918	0.90 (0.141–5.84)						
Cardiogenic shock	0.270	2 (0.58–6.89)						
STEMI	0.557	1.36 (0.48–3.83)						
NSTEMI	0.557	0.73 (0.26–2.06)						
R wave	0.138	0.40 (0.10–1.56)						
Diabetes	0.864	1.10 (0.29–3.10)						
Hypertension	0.519	0.70 (0.24–2.06)						
CKD	0.300	1.85 (0.58–5.90)						
Previous PCI	0.821	1.16 (0.28–4.82)						
RV dysfunction	0.217	0.24 (0.02–2.40)						
LVEDVi (ml/mq)	0.894	1.00 (0.96–1.05)						
LVEF at baseline (%)	0.048	0.91 (0.83–0.99)	0.064	0.89 (0.79–1)	0.012	0.86 (0.76–0.97)	0.013	0.85 (0.75–0.96)
Lactate ** (mmol/)	0.302	1.13 (0.89–1.44)						
CK-MB peak (U/L)	0.446	0.99 (0.97–1.02)						
Vasoactive drugs *	0.926	0.93 (0.21–3.99)						
LM disease	0.878	0.92 (0.33–2.57)						
LAD disease	0.835	1.14 (0.32–4.01)						
N° of critical vessels	1	1 (0.44–2.24)						
N° of treated vessels	0.951	0.98 (0.48–2.01)						
Functional CR	0.013	5.5 (1.4–21)	0.012	6.29 (1.49–26.1)				
Syntax score	0.363	0.98 (0.93–1.03)						
RI	0.031	13.5 (1.2–143.4)			0.013	33.2 (2.00–538.5)		
BCISJ pre	0.247	0.86 (0.67–1.11)						
BCISJ post	0.023	0.78 (0.60–0.96)					0.010	0.72 (0.56–0.92)

Model A included LVEF at baseline (%) and complete functional revascularization, model B included LVEF at baseline (%) and revascularization index, and model C included LVEF at baseline (%) and BCISJ post. BMI = body mass index; CR = complete revascularization; CKD = chronic kidney disease, abnormalities of kidney function (decreased glomerular filtration rate < 60 mL/min/1.73m^2^) or kidney structure (e.g., kidney transplantation); LM = left main; LAD = left anterior descending coronary artery; PCI = percutaneous coronary interventions; BCIS-JS = British Cardiovascular Intervention Society myocardial jeopardy score; CABG = coronary artery bypass graft; OHCA = out-of-hospital cardiac arrest; STEMI = ST-elevation myocardial infarction; NSTEMI = non-ST-elevation myocardial infarction; LVEDVi = left ventricular end-diastolic volume; RBBB = right bundle branch block; RV = right ventricle; CK-MB = creatine kinase-MB; RI = revascularization index; * norepinephrine, epinephrine, dopamine, dobutamine; alone or in combination; ** lactate upon admission.

**Table 5:** Univariate Cox regression analysis of the predictors of three-year mortality.

	*p*-Value	HR (95% C.I.)
Age (year)	0.332	1.03 (0.97–1.11)
Sex	0.563	1.63 (0.34–7.80)
BMI	0.746	1.02 (0.90–1.15)
OHCA	0.621	1.77 (0.21–14.40)
Cardiogenic shock	0.677	1.34 (0.33–5.37)
STEMI	0.512	1.56 (0.41–5.89)
NSTEMI	0.512	0.64 (0.17–2.42)
R wave	0.265	2.26 (0.54–9.51)
ST deviation	0.317	0.47 (0.10–2.05)
Diabetes	0.796	1.2 (0.30–4.81)
Hypertension	0.721	1.29 (0.32–5.16)
CKD	0.88	1.10 (0.28–4.44)
Previous PCI	0.508	1.70 (0.35–8.23)
RV dysfunction	0.971	0.96(0.10–8.67)
LVEDVi(ml/mq)	0.132	0.97 (0.92–1.01)
LVEF at baseline (%)	0.992	1.00 (0.90–1.11)
Lactate, admission (mmol/)	0.230	1.14 (0.92–1.41)
CK-MB peak (U/L)	0.665	1.00 (0.98–1.03)
Vasoactive drugs *	0.432	1.92 (0.38–9.80)
LM disease	0.339	0.51 (0.13–2.05)
LAD disease	0.371	0.39 (0.05–3.11)
Number of critical vessels	0.281	0.61 (0.25–1.49)
Number of treated vessels	0.765	1.16 (0.46–2.89)
Functional complete revascularization	0.975	0.98 (0.23–4.11)
Syntax score	0.067	1.066 (0.996–1.141)
Revascularization index	0.856	0.78 (0.06–10.99)
BCISJ pre	0.154	1.43 (0.88–2.33)
BCISJ post	0.671	1.05 (0.83–34)
**Delta EF ≥ 5%**	0.038	5.3 (1.1–25.7)
**LVEF at follow-up (%)**	**0.014**	**0.89 (0.82–0.98)**

BMI = body mass index; CKD = chronic kidney disease, abnormalities of kidney function (decreased glomerular filtration rate < 60 mL/min/1.73m^2^) or kidney structure (e.g., kidney transplantation); LM = left main; LAD = left anterior descending coronary artery; PCI = percutaneous coronary interventions; BCIS-JS = British Cardiovascular Intervention Society myocardial jeopardy score; CABG = coronary artery bypass graft; OHCA = out-of-hospital cardiac arrest; STEMI = ST-elevation myocardial infarction; NSTEMI = non-ST-elevation myocardial infarction; LVEF = left ventricular ejection fraction; LVEDVi = left ventricular end diastolic volume; RBBB = right bundle branch block; RV = right ventricle; CK-MB = creatine kinase-MB. * norepinephrine, epinephrine, dopamine, dobutamine; alone or in combination.
